# *N*-[(1,3-Benzodioxol-5-yl)meth­yl]-4-methyl­benzamide: an analogue of capsaicin

**DOI:** 10.1107/S1600536813002201

**Published:** 2013-02-02

**Authors:** Stella H. Maganhi, Mariana C. F. C. B. Damião, Maurício T. Tavares, Roberto Parise Filho

**Affiliations:** a Departmento de Química, Universidade Federal de São Carlos, 13565-905 São Carlos, SP, Brazil; b Departmento de Farmacia, Universidade de São Paulo, 05508-000 São Paulo, SP, Brazil

## Abstract

In the title compound, C_16_H_15_NO_3_, the five-membered 1,3-dioxole ring is in an envelope conformation with the methyl­ene C atom as the flap atom [lying 0.202 (3) Å out of the plane formed by the other four atoms]. The benzene ring makes a dihedral angle of 84.65 (4)° with the best least-squares plane through the 1,3-benzodioxole fused-ring system, which substitutes the 2-methoxyphenol moiety in capsaicin. In the crystal, mol­ecules are connected into a zigzag supra­molecular chain along the *c-*axis direction by N—H⋯O hydrogen bonds. These chains are connected into a layer in the *ac* plane by C—H⋯π inter­actions.

## Related literature

For the biological activity of capsaicin, see: Okamoto *et al.* (2011 ▶). For ring conformational analysis, see: Cremer & Pople (1975 ▶).
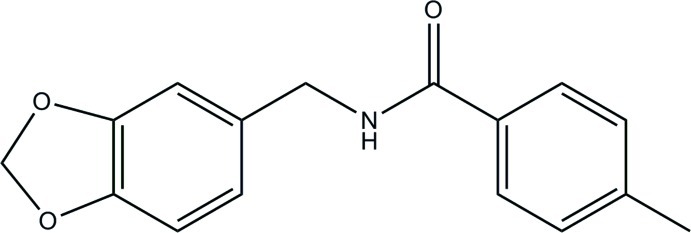


## Experimental

### 

#### Crystal data

C_16_H_15_NO_3_
*M* *_r_* = 269.29
Monoclinic, 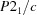

*a* = 4.9810 (2) Å
*b* = 26.652 (1) Å
*c* = 10.0545 (3) Å
β = 92.139 (2)°
*V* = 1333.84 (8) Å^3^
*Z* = 4
Mo *K*α radiation
μ = 0.09 mm^−1^
*T* = 290 K
0.33 × 0.24 × 0.16 mm

#### Data collection

Bruker APEXII CCD area-detector diffractometer
Absorption correction: numerical (*SADABS*; Sheldrick, 1996 ▶) *T*_min_ = 0.940, *T*_max_ = 0.951
4550 measured reflections
2602 independent reflections
1698 reflections with *I* > 2σ(*I*)
*R*_int_ = 0.023

#### Refinement

*R*[*F* ^2^ > 2σ(*F* ^2^)] = 0.050
*wR*(*F* ^2^) = 0.149
*S* = 1.03
2602 reflections
182 parameters
H-atom parameters constrained
Δρ_max_ = 0.21 e Å^−3^
Δρ_min_ = −0.20 e Å^−3^

### 

Data collection: *COLLECT* (Nonius, 1999 ▶); cell refinement: *SCALEPACK* (Otwinowski & Minor, 1997 ▶); data reduction: *DENZO* (Otwinowski & Minor, 1997 ▶) and *SCALEPACK*; program(s) used to solve structure: *SIR97* (Altomare *et al.*, 1999 ▶); program(s) used to refine structure: *SHELXL97* (Sheldrick, 2008 ▶); molecular graphics: *ORTEP-3 for Windows* (Farrugia, 2012 ▶); software used to prepare material for publication: *Marvinsketch* (Chemaxon, 2010 ▶) and *publCIF* (Westrip, 2010 ▶).

## Supplementary Material

Crystal structure: contains datablock(s) global, I. DOI: 10.1107/S1600536813002201/tk5191sup1.cif

Structure factors: contains datablock(s) I. DOI: 10.1107/S1600536813002201/tk5191Isup2.hkl

Supplementary material file. DOI: 10.1107/S1600536813002201/tk5191Isup3.cml

Additional supplementary materials:  crystallographic information; 3D view; checkCIF report

## Figures and Tables

**Table 1:** Hydrogen-bond geometry (Å, °) *Cg*1 and *Cg*2 are the centroids of the C1–C6 and C10–C15 rings, respectively.

*D*—H⋯*A*	*D*—H	H⋯*A*	*D*⋯*A*	*D*—H⋯*A*
N1—H1*N*1⋯O3^i^	0.91	2.08	2.958 (2)	162
C7—H7*A*⋯*Cg*1	0.97	2.74	3.603 (3)	149
C16—H16*C*⋯*Cg*2	0.96	2.96	3.829 (2)	151

Symmetry code: (i) 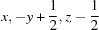.

**Fig. 1.**
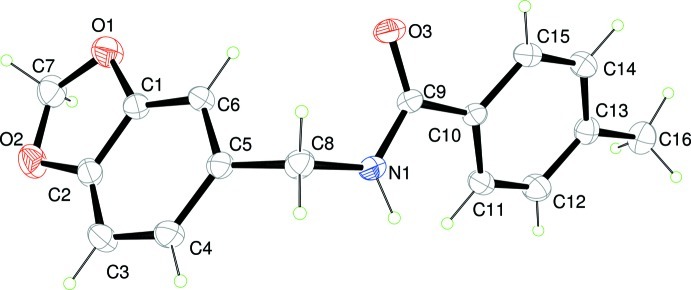
The molecular structure of (I) showing atom labelling scheme and displacement ellipsoids at the 50% probability level (arbitrary spheres for the H atoms).

## supplementary crystallographic information

### Comment

Capsaicin is the main pungent compound found in chilli peppers of the genus *Capsicum* and is found to exert multiple pharmacological and physiological effects, including analgesic, anti-cancer, anti-inflammatory, anti-oxidant and anti-obesity (Okamoto *et al.* 2011). This makes capsaicin an excellent scaffold for the rational design of analogues with better biological activity. The title compound (I) is a capsaicin-like derivative where the 2-methoxyphenol ring was substituted with a benzodioxol ring and the amide aliphatic chain was replaced with a 4-methylbenzoyl group. As suitable crystals were obtained from its hexane solution, a crystal structure determination of (I) was undertaken.

In (I), Fig. 1, the 1,3-dioxole five membered ring is in an envelope configuration with the C7 atom lying 0.202 (3) Å out of the plane formed by the other four atoms. The ring puckering parameters are q_2_ = 0.128 (2) Å and φ_2_ = 149.9 (9)° (Cremer & Pople, 1975).

The crystal packing of (I), Table 1, is sustained by N—H···O and C—H···π interactions, leading to supramolecular layers in the *ac* plane.

### Experimental

1-(1,3-Benzodioxol-5-yl)methanamine (0.755 g, 5 mmol) was dissolved in chloroform (20 ml) and then triethylamine (411 µ*L*, 5.5 mmol) and DMF (40 µ*L*, 0,5 mmol) were added. The mixture was stirred for 30 min under argon atmosphere. 4-Methylbenzoyl chloride (661 µ*L*, 5 mmol) was added in portions and stirred over 24 h. The organic layer was washed with 5% HCl aqueous solution, water, brine and dried over anhydrous Na_2_SO_4_. The solvent was removed under high vacuum and the title compound was obtained after recrystallization from hot hexane. Analytical data: white solid, yield 71.4° (0.96 g, 3.57 mmol). ^1^H-NMR NMR (300 MHz, DMSO-d6, ppm): δ 8.87 (1*H*, bt, J = 5.8 Hz, 9-NH), 7.79 (2*H*, d, J = 8.2 Hz,12, 13-ArH), 7.27 (2*H*, d, J = 8.2 Hz, 14, 15-ArH), 6.83 (3*H*, m, 4, 5, 7-ArH), 5.98 (2*H*, s, 1-OCH_2_O), 4.38 (2*H*, d, J = 6.0 Hz, 8-CH_2_), 2.36 (3*H*, s, 15-CH_3_). ^13^C NMR (75 MHz, DMSO-d6, ppm) δ: 165.9 (C9), 147.18 (C2), 145.98 (C1), 141.02 (C13), 133.68 (C10), 131.67 (C5), 128.77 (C12, C14), 127.21 (C11, C15), 120.42 (C4), 107.94 (C3), 107.92 (C6), 100.74 (C7), 42.36 (C8), 20.89 (C16); Anal. calcd. for: C_16_H_15_NO_3_ (269.11): C 71.36, H 5.61, N 5.20, found: C 71.30, H 5.74, N 5.17, mp: 404.7–405.2 K.

### Refinement

The H atoms were geometrically placed (C—H = 0.93–0.97 Å) and refined as riding with *U_iso_*(H) = 1.2–1.5*U_eq_*(C). The N—H H atom was located in a difference map, fixed in this position with *U_iso_*(H) = 1.2*U_eq_*(N).

### Crystal data

**Table:**

C_16_H_15_NO_3_	*F*(000) = 568
*M**_r_* = 269.29	*D*_x_ = 1.341 Mg m^−^^3^
Monoclinic, *P*2_1_/*c*	Mo *K*α radiation, λ = 0.71073 Å
Hall symbol: -P 2ybc	Cell parameters from 5124 reflections
*a* = 4.9810 (2) Å	θ = 3.1–27.5°
*b* = 26.652 (1) Å	µ = 0.09 mm^−^^1^
*c* = 10.0545 (3) Å	*T* = 290 K
β = 92.139 (2)°	Prism, colourless
*V* = 1333.84 (8) Å^3^	0.33 × 0.24 × 0.16 mm
*Z* = 4	

### Data collection

**Table:**

Bruker APEXII CCD area-detector diffractometer	2602 independent reflections
Radiation source: fine-focus sealed tube	1698 reflections with *I* > 2σ(*I*)
Graphite monochromator	*R*_int_ = 0.023
Detector resolution: 0 pixels mm^-1^	θ_max_ = 26.0°, θ_min_ = 3.1°
φ and ω scans	*h* = −6→6
Absorption correction: numerical (*SADABS*; Sheldrick, 1996)	*k* = −32→30
*T*_min_ = 0.940, *T*_max_ = 0.951	*l* = −12→12
4550 measured reflections	

### Refinement

**Table:**

Refinement on *F*^2^	Primary atom site location: structure-invariant direct methods
Least-squares matrix: full	Secondary atom site location: difference Fourier map
*R*[*F*^2^ > 2σ(*F*^2^)] = 0.050	Hydrogen site location: inferred from neighbouring sites
*wR*(*F*^2^) = 0.149	H-atom parameters constrained
*S* = 1.03	*w* = 1/[σ^2^(*F*_o_^2^) + (0.0859*P*)^2^ + 0.0674*P*] where *P* = (*F*_o_^2^ + 2*F*_c_^2^)/3
2602 reflections	(Δ/σ)_max_ < 0.001
182 parameters	Δρ_max_ = 0.21 e Å^−^^3^
0 restraints	Δρ_min_ = −0.20 e Å^−^^3^

### Special details

**Table:**

Geometry. All s.u.'s (except the s.u. in the dihedral angle between two l.s. planes) are estimated using the full covariance matrix. The cell s.u.'s are taken into account individually in the estimation of s.u.'s in distances, angles and torsion angles; correlations between s.u.'s in cell parameters are only used when they are defined by crystal symmetry. An approximate (isotropic) treatment of cell s.u.'s is used for estimating s.u.'s involving l.s. planes.
Refinement. Refinement of *F*^2^ against ALL reflections. The weighted *R*-factor *wR* and goodness of fit *S* are based on *F*^2^, conventional *R*-factors *R* are based on *F*, with *F* set to zero for negative *F*^2^. The threshold expression of *F*^2^ > 2σ(*F*^2^) is used only for calculating *R*-factors(gt) *etc*. and is not relevant to the choice of reflections for refinement. *R*-factors based on *F*^2^ are statistically about twice as large as those based on *F*, and *R*- factors based on ALL data will be even larger.

### Fractional atomic coordinates and isotropic or equivalent isotropic displacement parameters (Å^2^)

**Table:**

	*x*	*y*	*z*	*U*_iso_*/*U*_eq_	
O1	−0.0219 (3)	0.06137 (6)	0.88685 (15)	0.0754 (5)	
O2	−0.0295 (3)	0.00662 (6)	0.71012 (17)	0.0797 (5)	
O3	0.4080 (3)	0.24272 (5)	0.87268 (12)	0.0634 (4)	
N1	0.4208 (3)	0.22490 (6)	0.65426 (14)	0.0496 (4)	
H1N1	0.3768	0.2357	0.5702	0.060*	
C1	0.1365 (4)	0.08000 (7)	0.78849 (19)	0.0525 (5)	
C2	0.1354 (4)	0.04694 (8)	0.6838 (2)	0.0585 (5)	
C3	0.2867 (5)	0.05485 (8)	0.5769 (2)	0.0733 (6)	
H3	0.2878	0.0322	0.5065	0.088*	
C4	0.4407 (4)	0.09851 (8)	0.5771 (2)	0.0649 (6)	
H4	0.5483	0.1047	0.5053	0.078*	
C5	0.4398 (3)	0.13305 (7)	0.68029 (18)	0.0503 (5)	
C6	0.2825 (3)	0.12319 (7)	0.79020 (18)	0.0505 (5)	
H6	0.2782	0.1453	0.8616	0.061*	
C7	−0.1575 (5)	0.01955 (9)	0.8290 (2)	0.0740 (7)	
H7A	−0.3444	0.0279	0.8094	0.089*	
H7B	−0.1510	−0.0086	0.8903	0.089*	
C8	0.5961 (4)	0.18139 (7)	0.6730 (2)	0.0556 (5)	
H8A	0.7042	0.1856	0.7545	0.067*	
H8B	0.7169	0.1795	0.5997	0.067*	
C9	0.3359 (3)	0.25214 (7)	0.75640 (17)	0.0467 (5)	
C10	0.1449 (3)	0.29384 (7)	0.72454 (16)	0.0440 (4)	
C11	−0.0105 (4)	0.29652 (7)	0.60682 (19)	0.0547 (5)	
H11	0.0065	0.2719	0.5422	0.066*	
C12	−0.1888 (4)	0.33522 (8)	0.5853 (2)	0.0620 (6)	
H12	−0.2918	0.3360	0.5062	0.074*	
C13	−0.2198 (4)	0.37300 (7)	0.6775 (2)	0.0535 (5)	
C14	−0.0639 (4)	0.37058 (8)	0.7936 (2)	0.0598 (5)	
H14	−0.0779	0.3958	0.8570	0.072*	
C15	0.1126 (4)	0.33142 (8)	0.81753 (18)	0.0576 (5)	
H15	0.2117	0.3303	0.8977	0.069*	
C16	−0.4161 (4)	0.41514 (8)	0.6534 (2)	0.0710 (6)	
H16A	−0.3634	0.4345	0.5782	0.106*	
H16B	−0.4180	0.4363	0.7307	0.106*	
H16C	−0.5924	0.4016	0.6358	0.106*	

### Atomic displacement parameters (Å^2^)

**Table:**

	*U*^11^	*U*^22^	*U*^33^	*U*^12^	*U*^13^	*U*^23^
O1	0.0884 (10)	0.0677 (10)	0.0708 (10)	−0.0258 (8)	0.0130 (8)	−0.0058 (8)
O2	0.0972 (11)	0.0531 (9)	0.0875 (12)	−0.0117 (8)	−0.0135 (9)	−0.0127 (8)
O3	0.0857 (10)	0.0681 (10)	0.0363 (8)	0.0119 (8)	−0.0003 (6)	0.0050 (7)
N1	0.0628 (9)	0.0497 (9)	0.0366 (8)	0.0042 (7)	0.0049 (7)	0.0051 (7)
C1	0.0582 (11)	0.0506 (12)	0.0482 (11)	0.0027 (9)	−0.0053 (9)	−0.0010 (9)
C2	0.0697 (13)	0.0469 (11)	0.0577 (13)	0.0060 (10)	−0.0126 (10)	−0.0048 (10)
C3	0.1024 (17)	0.0590 (14)	0.0577 (14)	0.0133 (13)	−0.0077 (12)	−0.0177 (11)
C4	0.0821 (14)	0.0646 (14)	0.0486 (12)	0.0180 (11)	0.0101 (10)	−0.0022 (10)
C5	0.0545 (11)	0.0504 (11)	0.0459 (11)	0.0121 (9)	0.0000 (8)	0.0027 (9)
C6	0.0592 (11)	0.0481 (11)	0.0440 (11)	0.0028 (9)	0.0003 (8)	−0.0066 (9)
C7	0.0766 (14)	0.0614 (14)	0.0827 (17)	−0.0153 (12)	−0.0136 (13)	−0.0005 (12)
C8	0.0553 (11)	0.0590 (12)	0.0530 (11)	0.0067 (10)	0.0089 (8)	0.0053 (9)
C9	0.0559 (10)	0.0482 (11)	0.0361 (10)	−0.0035 (8)	0.0040 (8)	0.0042 (8)
C10	0.0507 (10)	0.0452 (10)	0.0365 (9)	−0.0044 (8)	0.0069 (7)	0.0020 (8)
C11	0.0632 (11)	0.0533 (12)	0.0474 (11)	0.0016 (9)	−0.0009 (9)	−0.0075 (9)
C12	0.0610 (12)	0.0660 (14)	0.0582 (13)	0.0061 (11)	−0.0090 (9)	−0.0025 (11)
C13	0.0481 (10)	0.0524 (12)	0.0605 (12)	−0.0026 (9)	0.0088 (9)	0.0040 (10)
C14	0.0707 (13)	0.0547 (13)	0.0544 (12)	0.0039 (10)	0.0093 (10)	−0.0095 (10)
C15	0.0712 (13)	0.0598 (13)	0.0417 (11)	0.0020 (10)	−0.0008 (9)	−0.0037 (9)
C16	0.0593 (13)	0.0664 (14)	0.0879 (17)	0.0101 (11)	0.0121 (11)	0.0065 (12)

### Geometric parameters (Å, º)

**Table:**

O1—C1	1.380 (2)	C7—H7B	0.9700
O1—C7	1.417 (3)	C8—H8A	0.9700
O2—C2	1.384 (2)	C8—H8B	0.9700
O2—C7	1.418 (3)	C9—C10	1.490 (3)
O3—C9	1.2358 (19)	C10—C15	1.384 (3)
N1—C9	1.339 (2)	C10—C11	1.392 (3)
N1—C8	1.459 (2)	C11—C12	1.373 (3)
N1—H1N1	0.9124	C11—H11	0.9300
C1—C6	1.361 (3)	C12—C13	1.382 (3)
C1—C2	1.372 (3)	C12—H12	0.9300
C2—C3	1.352 (3)	C13—C14	1.380 (3)
C3—C4	1.394 (3)	C13—C16	1.503 (3)
C3—H3	0.9300	C14—C15	1.380 (3)
C4—C5	1.387 (3)	C14—H14	0.9300
C4—H4	0.9300	C15—H15	0.9300
C5—C6	1.403 (3)	C16—H16A	0.9600
C5—C8	1.508 (3)	C16—H16B	0.9600
C6—H6	0.9300	C16—H16C	0.9600
C7—H7A	0.9700		
			
C1—O1—C7	105.39 (16)	C5—C8—H8A	109.2
C2—O2—C7	105.13 (16)	N1—C8—H8B	109.2
C9—N1—C8	122.43 (15)	C5—C8—H8B	109.2
C9—N1—H1N1	117.9	H8A—C8—H8B	107.9
C8—N1—H1N1	119.5	O3—C9—N1	121.77 (17)
C6—C1—C2	122.7 (2)	O3—C9—C10	121.03 (16)
C6—C1—O1	127.92 (18)	N1—C9—C10	117.19 (15)
C2—C1—O1	109.40 (18)	C15—C10—C11	117.55 (17)
C3—C2—C1	121.5 (2)	C15—C10—C9	118.96 (16)
C3—C2—O2	128.7 (2)	C11—C10—C9	123.47 (17)
C1—C2—O2	109.73 (19)	C12—C11—C10	120.57 (18)
C2—C3—C4	116.89 (19)	C12—C11—H11	119.7
C2—C3—H3	121.6	C10—C11—H11	119.7
C4—C3—H3	121.6	C11—C12—C13	121.98 (18)
C5—C4—C3	122.5 (2)	C11—C12—H12	119.0
C5—C4—H4	118.7	C13—C12—H12	119.0
C3—C4—H4	118.7	C14—C13—C12	117.42 (18)
C4—C5—C6	118.83 (19)	C14—C13—C16	120.94 (19)
C4—C5—C8	120.96 (17)	C12—C13—C16	121.64 (18)
C6—C5—C8	120.17 (17)	C13—C14—C15	121.17 (18)
C1—C6—C5	117.52 (17)	C13—C14—H14	119.4
C1—C6—H6	121.2	C15—C14—H14	119.4
C5—C6—H6	121.2	C14—C15—C10	121.30 (17)
O1—C7—O2	108.35 (18)	C14—C15—H15	119.4
O1—C7—H7A	110.0	C10—C15—H15	119.4
O2—C7—H7A	110.0	C13—C16—H16A	109.5
O1—C7—H7B	110.0	C13—C16—H16B	109.5
O2—C7—H7B	110.0	H16A—C16—H16B	109.5
H7A—C7—H7B	108.4	C13—C16—H16C	109.5
N1—C8—C5	112.19 (14)	H16A—C16—H16C	109.5
N1—C8—H8A	109.2	H16B—C16—H16C	109.5
			
C7—O1—C1—C6	172.16 (19)	C9—N1—C8—C5	93.3 (2)
C7—O1—C1—C2	−9.7 (2)	C4—C5—C8—N1	108.75 (19)
C6—C1—C2—C3	1.8 (3)	C6—C5—C8—N1	−68.9 (2)
O1—C1—C2—C3	−176.41 (17)	C8—N1—C9—O3	2.1 (3)
C6—C1—C2—O2	179.79 (16)	C8—N1—C9—C10	−176.54 (15)
O1—C1—C2—O2	1.6 (2)	O3—C9—C10—C15	20.0 (3)
C7—O2—C2—C3	−174.9 (2)	N1—C9—C10—C15	−161.38 (17)
C7—O2—C2—C1	7.3 (2)	O3—C9—C10—C11	−158.13 (18)
C1—C2—C3—C4	−0.9 (3)	N1—C9—C10—C11	20.5 (3)
O2—C2—C3—C4	−178.44 (19)	C15—C10—C11—C12	−0.1 (3)
C2—C3—C4—C5	−0.7 (3)	C9—C10—C11—C12	178.03 (17)
C3—C4—C5—C6	1.5 (3)	C10—C11—C12—C13	0.6 (3)
C3—C4—C5—C8	−176.20 (18)	C11—C12—C13—C14	0.0 (3)
C2—C1—C6—C5	−1.0 (3)	C11—C12—C13—C16	−179.77 (18)
O1—C1—C6—C5	176.87 (17)	C12—C13—C14—C15	−1.1 (3)
C4—C5—C6—C1	−0.6 (3)	C16—C13—C14—C15	178.62 (18)
C8—C5—C6—C1	177.12 (16)	C13—C14—C15—C10	1.7 (3)
C1—O1—C7—O2	14.2 (2)	C11—C10—C15—C14	−1.1 (3)
C2—O2—C7—O1	−13.3 (2)	C9—C10—C15—C14	−179.27 (17)

### Hydrogen-bond geometry (Å, º)

Cg1 and Cg2 are the centroids of the C1–C6 and C10–C15 rings, respectively.

**Table:**

*D*—H···*A*	*D*—H	H···*A*	*D*···*A*	*D*—H···*A*
N1—H1*N*1···O3^i^	0.91	2.08	2.958 (2)	162
C7—H7*A*···*Cg*1	0.97	2.74	3.603 (3)	149
C16—H16*C*···*Cg*2	0.96	2.96	3.829 (2)	151

Symmetry code: (i) *x*, −*y*+1/2, *z*−1/2.

## References

[bb1] Altomare, A., Burla, M. C., Camalli, M., Cascarano, G. L., Giacovazzo, C., Guagliardi, A., Moliterni, A. G. G., Polidori, G. & Spagna, R. (1999). *J. Appl. Cryst.* **32**, 115–119.

[bb2] Chemaxon (2010). *Marvinsketch.* http://www.chemaxon.com.

[bb3] Cremer, D. & Pople, J. A. (1975). *J. Am. Chem. Soc.* **97**, 1354–1358.

[bb4] Farrugia, L. J. (2012). *J. Appl. Cryst.* **45**, 849–854.

[bb5] Nonius (1999). *COLLECT* Nonius BV, Delft, The Netherlands.

[bb6] Okamoto, M., Irii, H., Tahara, Y., Ishii, H., Hirao, A., Udagawa, H., Hiramoto, M., Yasuda, K., Takanishi, A., Shibata, S. & Shimizu, I. (2011). *J. Med. Chem.* **54**, 6295–6304. 10.1021/jm200662c 21851089

[bb7] Otwinowski, Z. & Minor, W. (1997). *Methods in Enzymology*, Vol. 276, *Macromolecular Crystallography*, Part A, edited by C. W. Carter Jr & R. M. Sweet, pp. 307–326. New York: Academic Press.

[bb8] Sheldrick, G. M. (1996). *SADABS* University of Göttingen, Germany.

[bb9] Sheldrick, G. M. (2008). *Acta Cryst.* A**64**, 112–122. 10.1107/S0108767307043930 18156677

[bb10] Westrip, S. P. (2010). *J. Appl. Cryst.* **43**, 920–925.

